# The emergence of ‘Transeurasian’ language families in Northeast Asia as viewed from archaeological evidence

**DOI:** 10.1017/ehs.2021.49

**Published:** 2022-01-06

**Authors:** Kazuo Miyamoto

**Affiliations:** Faculty of Humanities, Kyushu University, 744 Motooka, Nishi-ku, Fukuoka, 819-0395, Japan

**Keywords:** Proto-Japonic language, Proto-Koreanic language, Jomon culture, Yayoi culture, Mumun culture, Pianpu culture, pottery production technique, the Yan state, rolled rim vessel

## Abstract

From a linguistic standpoint, Proto-Japonic and Proto-Koreanic are assumed to have split off the Transeurasian languages in southern Manchuria. The linguistic idea that Proto-Japonic came earlier than Proto-Koreanic in the chronological scheme means that the Proto-Japonic language first entered the Korean Peninsula, and from there spread to the Japanese archipelago at the beginning of the Yayoi period, around the ninth century BC, while the arrival of Proto-Koreanic in southern Korea is associated with the spread of the rolled rim vessel culture around the fifth century BC. The genealogical sequence of the Pianpu, Mumun and Yayoi cultures, which shared the same pottery production techniques, indicates the spread of Proto-Japonic. On the other hand, migrants moved from Liaodong to the Korean Peninsula and established the rolled rim vessel culture. This population movement was probably due to social and political reasons as the Yan state enlarged its territory eastward. The Proto-Koreanic of the rolled rim vessel culture later spread to the Korean Peninsula and gradually drove out Proto-Japonic, becoming the predecessor of the Koreanic. In this paper, I examine the spread of Proto-Japonic and Proto-Koreanic in Northeast Asia based on archaeological evidence, focusing especially on the genealogy of pottery styles and pottery production techniques.

## Graphical abstract

**Figure:**
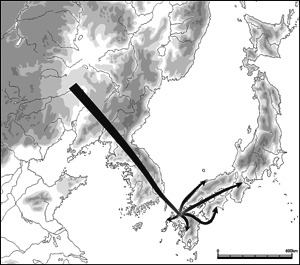

**Social media summary:** From a linguistic standpoint, Proto-Japonic and Proto-Koreanic are assumed to have split off the Transeurasian languages in southern Manchuria. In this paper, I examine the spread of Proto-Japonic and Proto-Koreanic in Northeast Asia based on archaeological evidence, focusing especially on the genealogy. The genealogical sequence of the Pianpu, Mumun and Yayoi cultures, which shared the same pottery production techniques, indicates the spread of Proto-Japonic. On the other hand, migrants of Proto-Koreanic moved from Liaodong to the Korean Peninsula and established the rolled rim vessel culture. The Proto-Koreanic of the rolled rim vessel culture later spread to the Korean Peninsula and gradually drove out Proto-Japonic, becoming the predecessor of the Koreanic.

## 1. Introduction

The view that language dispersals in prehistory are related to the spread of agriculture with human migration is a well-known hypothesis first put forward by Colin Renfrew (1987), suggesting that the spread of Indo-European languages accompanied the diffusion of agriculture. He demonstrated archaeologically that agricultural people, whose homeland was in southern central Anatolia, spread into Greece by 7000–6500 BC. This dispersal continued until they reached Europe to form a subgroup of the Indo-European languages. In addition, he also showed that there was exchange between migrant agricultural people and indigenous Mesolithic people over the course of 3000 years: from 7000 BC when the Indo-European languages spread to Greece to 4000 BC when they spread to the British Isles (Renfrew, 1987, 1999). This hypothesis is often called the ‘Farming-Language Dispersal Hypothesis’ and is tied to demographic growth resulting from agriculture surplus.

Peter Bellwood also hypothesised that the spread of agriculture was similarly linked to the spread of languages in East Asia (Bellwood, 2005). According to this hypothesis, the Sino-Tibetan languages originated alongside millet-based agriculture in the middle basin of the Yellow River, whereas the Hmong-Mien language family, associated with rice-based agriculturalists in the middle basin of the Yangtze River, moved into Southeast Asia to form the Austroasiatic language family. In addition, the Tai language family, spoken by people who practised rice agriculture in southern China, developed into part of the Austroasiatic language family in Southeast Asia. Additionaly Austronesian spread southward from Taiwan into Indonesia and Oceania.

Meanwhile, the following five linguistic families are traditionally categorised as ‘Altaic’: Japonic, Koreanic, Tungusic, Mongolic and Turkic. In recent years, the term ‘Transeurasian languages’ has been used to describe these five families, referring geographically to a large language group stretching from the Pacific in the east to the Baltic, the Black Sea and the Mediterranean in the west (Robbeets & Savelyev, 2020). Janhunen suggested that the homelands of the Transeurasian languages are situated in present-day eastern Mongolia, southern Manchuria and Korea (Janhunen, 2010). Robbeets also supported the farming dispersal hypotheses of Renfrew, Bellwood and others as the driving force behind the dispersal of Transeurasian languages (Robbeets, 2020). She argued that Macro-Japonic was spoken on the Liaodong Peninsula and was transmitted to the Japanese archipelago with the spread of rice agriculture via the Korean Peninsula (Robbeets, 2020).

The author in contrast suggested that while Proto-Japonic was transmitted to the Japanese archipelago with the spread of rice agriculture from southern Korea, the spread of Proto-Japonic from the Liaodong district to the Korean Peninsula was not connected with the spread of rice agriculture (Miyamoto, 2016, 2019b). Tao Li also criticised the farming dispersal hypothesis as the driving force behind dispersal of Proto-Transeurasian languages, especially for that of Proto-Tungusic. He argued that there are uncertainties and limitations in the archeological evidence for the farming dispersal hypothesis in the dispersal of Proto-Transeurasian languages (Li, 2020). Tao Li also suggested a link between the spread of millet farming to the Russian Far East and Proto-Tungusic (Li et al., 2020).

Recently Hudson and Robbeets suggested that the spread of Proto-Macro-Koreanic on the Korean Peninsula from Liaodong district was associated with the spread of millet cultivation at ca. 3500 BC in the Middle Korean Neolithic (Hudson & Robbeets, 2020). However, Kim and Park mentioned that the introduction of millet did not appear to have had an impact on the Chulmun material culture of the Neolithic Korean Peninsula, and that the pottery styles of northeast China (Liaoxi and Liaodong districts) and the Korean Peninsula in the Neolithic are clearly different (Kim & Park, 2020).

The author has also hypothesised that there were four stages in the development of agriculture in Northeast Asia during prehistory (Miyamoto, 2014, 2015a, 2017, 2019a). The first stage involved the spread of millet agriculture from southern Manchuria to the Korean Peninsula and to the southern Russian Far East at around 3300 BC. The second stage was the spread of wet-rice agriculture from the Shandong Peninsula to the Liaodong Peninsula at around 2400 BC. The third stage was the spread of irrigated agriculture associated with new polished stone tools, including reaping knives and flat plano-convex stone adzes. The third stage saw the introduction of a third agricultural system consisting of wet (rice) and dry (millet, wheat, etc.) fields. This irrigated agriculture also spread from the Shandong Peninsula through the Liaodong Peninsula to the Korean Peninsula at around 1500 BC. Finally, the fourth stage involved the spread of irrigated agriculture to northern Kyushu, Japan, beginning around the ninth century BC (Miyamoto, 2018). This theory of a four-stage developmental process of the spread of agriculture in Northeast Asia is based on the reasons why some agricultural people moved into the lands of hunter–gatherer societies to cultivate cereals owing to population pressures brought on by cooler climatic conditions.

In this paper, the author reexamines the spread of Proto-Japonic and Proto-Koreanic in Northeast Asia based on archeological evidence, focusing especially on the genealogy of pottery styles and production techniques. The author suggests that the spread of agriculture was not necessarily related to the dispersal of Proto-Japonic and Proto-Koreanic, except for the spread of agriculture from southern Korea to northern Kyushu with the spread of Proto- Japonic (Miyamoto, 2016, 2019b).

## 2. History of research on the Japonic and Koreanic languages

Some linguists regard the Japonic and Koreanic languages as being affiliated with Altaic (Ruhlen, 1987). These are relatively shallow languages, with Japonic and Koreanic splitting out from Macro-Tungusic family in the chronological scheme (Unger, 2009). The underlying linguistic interaction between Korean and Japanese took place at a time when Japonic (Para-Japonic) was still spoken in parts of Korea (Janhunen, 2005). The Koguryŏ toponymic data indicates that a language cognate to Japonic was spoken on the Korean Peninsula (Whitman, 2011). In addition, Japonic-sourced toponyms included in place names before the eighth century AD are typically distributed in the central and northern areas of the Yalu River, primarily in the district of Koguryŏ (Endo, 2021). Therefore, Japonic languages are hypothesised to have originally been used on the Korean Peninsula, although, some scholars have suggested that only some pockets of people in the southern tip of the Korean Peninsula spoke Japonic (Whitman, 2011; Vovin, 2013).

Lee and Hasegawa estimate a date of 2182 BP for the ancestor of Proto-Japonic using Bayesian phylogenic analysis based on lexical data from 59 Japonic varieties (Lee & Hasegawa, 2011). Because Japonic was spoken on the Korean Peninsula, it is believed that Japonic was spoken by both the Mumun culture on the Korean Peninsula and the Yayoi culture in the Japanese archipelago (Whitman, 2011). Furthermore, philologists believe that Japonic spread from the Korean Peninsula to the Japanese archipelago at the beginning of the Yayoi period through demic diffusion (Whitman, 2011; Vovin, 2013; Unger, 2014; Hudson et al., 2020). Therefore, it is probable that Japonic was spoken on the Japanese archipelago during the Yayoi period from around the ninth century BC to the third century AD.

Marshall Unger suggested that if the homeland of the Macro-Tungusic family, of which Proto-Japonic and Proto-Koreanic was one branch, was the area around the Bohai Gulf from Shandong to Liaoning, it meant that Proto-Tungusic speakers moved north and northeast finally into eastern Siberia, while Proto-Koreanic speakers split off and moved into southern Manchuria. He also concluded that Proto-Japonic speakers brought the knowledge of wet-field rice cultivation to the Korean Peninsula, and that Koreanic speakers moved into the Korean Peninsula and drove out the Proto-Japonic speakers (Unger, 2014). The Koreanic language is believed to have spread with the migration of people from the Liaoning district to the Korean Peninsula with the advent of the slender dagger (Korean type dagger) culture (Whitman, 2011).

Martine Robbeets and others assume that, with the spread of millet agriculture, Proto-Macro-Japonic came to be spoken in an area extending from the Liaodong Peninsula to the Korean Peninsula between 3500 and 1500 BC. Para-Japonic was spoken in the Korean Peninsula after 1500 BC with spread of rice agriculture via the Liaodong Peninsula from the Shandong Peninsula. Proto-Japonic split from Para-Japonic and spread to Kyushu to become the Yayoi culture with agriculture around 900 BC (Robbeets et al., 2020). Her interpretation suggests that the ancestral speakers of Japonic–Koreanic may have been located along the Bohai coast and on the Liaodong Peninsula in the fourth millennium BC. She used Bayesian inference to indicate that Japonic and Koreanic separated around 1847 BC (Robbeets & Bouckaert, 2018). Silla in the southern Korean Peninsula, of which the language was the direct ancestor of Middle and Contemporary Korean, superseded all previous languages by the seventh century AD at the latest.

According to linguistic research on Proto-Japonic and Proto-Koreanic, it is probable that Proto-Japonic was spoken in the Korean Peninsula, and the Proto-Koreanic, which subsequently split off from the Transeurasian Languages in southern Manchuria, spread to the Korean Peninsula. Proto-Japonic probably spread to Japan in the Yayoi period with rice agriculture, although one linguistic theory exists suggesting that Central Japanese was heavily influenced by Old Korean (possibly Paekche) during the Kofun and Asuka periods, from the fourth to the seventh centuries AD (Unger, 2009). This is because the beginning of the Yayoi culture was heavily influenced by the Mumun culture of the southern Korean Peninsula, and some migrants from this region are thought to have moved into northern Kyushu (Miyamoto, 2014, 2016, 2017, 2019a; Hudson et al., 2020). If Proto-Japonic spread to northern Kyushu from the southern Korean Peninsula at the beginning of the Yayoi culture with migrants, then we can assume that Proto-Japonic was spoken by the people of the Mumun culture.

## 3. Problems concerning the dispersal of languages in an archeological context

The physical anthropological differences between the Jomon and Yayoi in northern Kyushu are very clear, with Yayoi people in northern Kyushu being physically similar to continental (Mumun) people (Nakahashi & Nagai, 1989; Hudson et al., 2020). Migrants from southern Korea mixed with native Jomon people in northern Kyushu, and the resulting Yayoi people gradually became dominant in this area (Tanaka & Ozawa, 2001). At the beginning of the Yayoi culture, from the ninth to the sixth centuries BC in northern Kyushu, these people cultivated rice in paddies, a form of irrigated agriculture. They also developed new polished agricultural tools like reaping stone knives, new burial customs such as dolmens, and different pottery styles including necked jars (Tsubo) and proto-Itazuke jar pottery, a type of jar belonging to the Mumun culture in the southern Korean Peninsula. In addition, circular moated settlements and wooden coffin burials emerged in the Yusu 2 period.

The transitional time between the Jomon and Yayoi cultures in northern Kyushu can be divided into three phases, from the Final Jomon Kurokawa phase to the Early Yayoi Itazuke 1, resulting in the emergence of the Itazuke pottery style (Table 1). The emergence of the Yayoi, which is the Yusu 1 period in the Initial Yayoi period, is dated to between the ninth and eighth centuries BC based on charred rice grains, 842–806 cal. BC (medium data of four samples), found at Ukikunnden Shell Midden (Miyamoto, 2018). These phases are believed to represent the process of genetic mixing between migrants of the Mumun culture from southern Korea and the Jomon people in northern Kyushu (Tanaka & Ozawa, 2001).

**Table 1.** Chronology and cultural change in the transition from Jomon to Yayoi in northern Kyushu

Phase	Pottery	Language	Tools	Influence	Burials
Final Jomon	Kurokawa	Jomon		Konyol rim punctuates	
Initial Yayoi	Yusu 1	Jomon/Japonic	New tools	KorPen south coast	Dolmens
			Stone dagger type 1		
	Yusu 2	Jomon/Japonic	Stone dagger type 2	KorPen Hoseo/Yongnam	Wood coffins
Early Yayoi	Itazuke 1	Japonic	Stone dagger type 2		Wood coffins

During the first period of cooling at around the 13th to 11th centuries BC, in the Kurokawa Jomon phase (Miyamoto, 2017), there is a period of brief contact between southern Korea and northern Kyushu. Yoshiyuki Tanaka (1991) proposed that some migrants came from southern Korea to northern Kyushu during the Kurokawa period, based on archaeological evidence, such as a row of punctuations under the rim of Kurokawa pottery, which imitated the Konyol style pottery of Korea, and a reaping knife found at the Nukigawa Site (Table 1). This is also related to cooler climatic conditions dating to c. 1200 BC (Miyamoto, 2017). Rice probably spread to Kyushu by this time, if not earlier, as indicated by evidence from SEM silicon analysis on pottery (Obata & Kunikida, in press).

Kurokawa Jomon pottery was gradually transformed into Yusu pottery through the spread of banded deep bowl culture (different from the band-rim Mumun pottery culture), originating in the Eastern Seto (Inland Sea area or Kinki) area and gradually spreading westward to northern Kyushu, replacing the Kurokawa pottery of the Final Jomon as Yusu pottery of the Initial Yayoi. Yusu pottery consisted of a two-type series: a deep bowl based on Jomon types and a necked jar (Tsubo) based on the Mumun culture (Miyamoto, 2016, 2017). During Yusu 1 (c. ninth to eighth centuries BC) and Yusu 2 (c. seventh to sixth centuries BC), the Mumun culture spread from two different locations on the Korean Peninsula. The former spread from the Namgang River to the Karatsu and Itoshima Plains, and the latter spread from the lower basin of the Nagtonggang River to the Fukuoka Plains (Miyamoto, 2016, 2019a). Yusu 2 preceded the development of the Itazuke (Early Yayoi) pottery style on the Fukuoka Plains, its initial location (Miyamoto, 2016). Thus, around the sixth to fifth centuries BC, Itazuke jar pottery influenced by Mumun ceramics replaced the deep bowls of the Jomon during the Yusu period. In the following sections, the ‘transitional’ period refers to the Yusu 1 and Yusu 2 phases. Although, they are considered Yayoi because of the presence of wet-rice agriculture, full-fledged Yayoi culture did not coalesce until Itazuke 1. The transitional period between Jomon and Early Yayoi in northern Kyushu took an estimated 300 years (Miyamoto, 2017).

**Figure 1.**
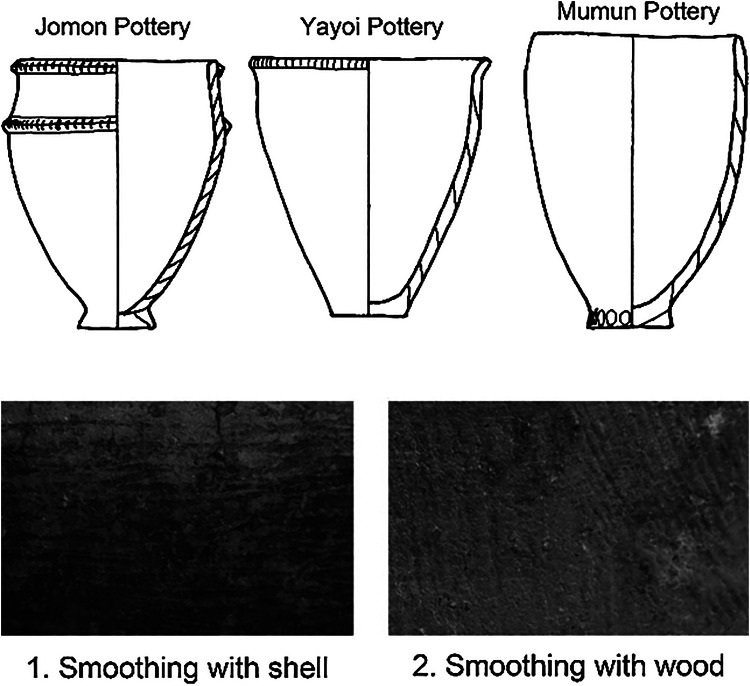
Differences in pottery production techniques between the Jomon and Yayoi

Not only the styles and genealogy of pottery, but also the pottery production techniques, differ between the Yayoi and Jomon. Although the necked jar was added through the influence of the Mumun pottery culture during the Yusu 1 type period, the deep bowls of the Jomon were still in use during the transitional phase between the Jomon and Yayoi. Itazuke jar pottery was established during the Itazuke 1 period in the Early Yayoi (Miyamoto, 2016, 2017). At this time, pottery production techniques totally changed from the Jomon period (Yane, 1984, 1997; Misaka, 2014). Relatively wide clay slabs were used to make Yayoi pottery, in contrast to the relatively thin clay coils used in Jomon pottery (Figure 1). The slabs of Yayoi pottery were attached on the outer surface of the previous slab, while the clay coils of Jomon pottery were laid up attached to the inner surface of the vessel. The surface of Yayoi pottery was smoothed using the edge of a piece of wood, while a shell was used in the Final Jomon to perform this task (Figure 1). Yayoi pottery was fired in a less elaborate clay kiln on the ground, but Jomon pottery was fired in an open space (Kobayashi et al., 2000). These four attributes of different pottery production techniques clearly existed between Jomon and Yayoi pottery. The four attributes of new pottery techniques for Yayoi pottery were adopted from Mumun pottery (Yane, 1984, 1997; Misaka, 2014). It is interesting that new techniques for pottery production were not fully established in the Yusu type, which was firstly influenced by the Mumun culture, but in the Itazuke type after the transitional period between the Jomon and Yayoi. The Itazuke type pottery after the transitional period between Jomon and Yayoi ended up consisting of four attributes of new pottery production techniques.

Additional cultural changes beyond ceramic styles are also apparent in the transition from Jomon to Yayoi (Table 1). Rice paddies, new types of pottery styles and new forms of polished stone tools are found on the Fukuoka Plains in Yusu 1 after the Kurokawa period (Miyamoto, 2016, 2017, 2019a; Fujio, 2021). Later, circular moat settlements were established at the time of Yusu 2. New burial customs like wooden coffins started in the Fukuoka Plains around the time of Yusu 2 and Itazuke 1 (Miyamoto, 2016, 2017, 2019a). Cultural attributes were added gradually in the time period between Yusu 1 and Yusu 2. Although dolmens were established at the time of Yusu 1, their distributions were concentrated in the Karatsu and Itoshima Plains, including northwestern Kyushu. However, wooden coffins were introduced from the Korean Peninsula to the Fukuoka Plains and then spread eastward from there into other areas at the time of Yusu 2. Polished stone daggers accompanied the wooden coffins as grave goods. Differences in the time of introduction and distribution area between dolmens and wooden coffins, along with differences in the distribution area by type of polished stone daggers on the Korean Peninsula, indicate the dual spread of culture from the Korean Peninsula (Miyamoto, 2016, 2019a). Thus, the Yayoi culture was established with the dual diffusion of the Mumun culture (Table 1). By the time of Itazuke 1, an irrigated agricultural society had formed (Miyamoto, 2017; Fujio, 2021).

With the establishment of the Yayoi culture, the creation of the Itazuke pottery style signified the development of an independent farming society in the Japanese archipelago. The new farming people of the Yayoi in northern Kyushu moved into the Seto Inland Sea and Kinki area, where the new farming people and native Jomon people once again intermixed.

Archeological evidence provides answers to the question of how the Proto-Japonic language culture spread to northern Kyushu from the southern Korean Peninsula, which corresponds to the Mumun pottery culture in the Korean Peninsula. In the previous section, I explained how rice spread to northern Kyushu in the Kurokawa period, and how rice agriculture with rice paddies spread to northern Kyushu in the Yusu period. Cultural changes including dolmens, polished stone tools and a pottery style influenced by the Mumun culture emerged in Yusu 1. Therefore, by definition, we can state that the Yayoi culture started in the Yusu 1 period, dating to between the ninth and eighth centuries BC.

Can we, therefore, infer from this that Proto-Japonic spread to northern Kyushu in the Yusu 1 period with migrants from the Mumun culture? We know that new pottery types such as necked jars influenced by the Mumun culture appeared in Yusu 1 in northern Kyushu. However, a large number of the new type of necked jars and other Jomon styles of pottery in the Yusu 1 period were made using Jomon pottery production techniques. Yayoi style pottery, including necked jars and Itazuke jars, created using with the same pottery production technique as that of the Mumun pottery culture, emerged in the Itazuke 1 period, dating to between the sixth and fifth centuries BC (Miyamoto, 2018). The author's view is that Proto-Japonic replaced the Jomon language in northern Kyushu at this time.

In the Yusu 1 period, Jomon people migrated together with a handful of Mumun migrants in northern Kyushu (Tanaka & Ozawa, 2001), who created necked jars for preserving domesticated cereals and Jomon deep bowls for cooking. It is thought that they imitated the necked jars of the Mumun culture for agricultural life using Jomon production techniques. However, the pottery style and production techniques of the Mumun culture changed in the Itazuke 1 period. The author suggests that the Jomon people were taught the new pottery style and production techniques through the Proto-Japonic language, which replaced the Jomon language in northern Kyushu. After this time, in the Early Yayoi period, the new Itazuke pottery style and production techniques spread from northern Kyushu, centring on the Fukuoka Plains, to the whole of western Japan, including northwestern Kyushu, the Seto Inland Sea area and the Kinki area. In this case, it is interesting to note that even the Yayoi people of northwestern Kyushu, who were genetically descended from the Jomon people, were able to make the same Yayoi pottery, as is the case with Itazuke pottery. Physical anthropological analysis has identified human bones belonging to the Yayoi people of northwestern Kyushu as being identical to those of the Jomon people (Nakahashi & Nagai, 1989). Recent DNA research shows that two individuals of northwestern people dating to the end of Yayoi period, first to second century AD, at Shimoyamatoyama site have the genome of both Jomon and immigrant Yayoi people (Shinoda et al., 2019). However, another DNA study indicates that the early Yayoi people of northwestern Kyushu at Otomo site were still the Jomon people (Kanazawa et al., 2021). Although the early Yayoi people of northwestern Kyushu did not genetically migrate with the Mumun people, they could make Yayoi pottery based on the pottery production techniques of the Mumun culture. This is because they were able to learn about the production techniques of Yayoi pottery through the Proto-Japonic language.

## 4. Archaeological explanation for the diffusion theory of the Proto-Japonic language

If the hypothesis that Proto-Japonic spread to northern Kyushu at the beginning of the Yayoi culture and fully replaced the Jomon language in the Itazuke 1 period is true, the question we must now ask is how and from where the Proto-Japonic language culture, which corresponds to the Mumun pottery culture, entered the Korean Peninsula. In order to resolve this issue, we choose to focus on pottery production techniques to explain cultural contact that is unrelated to the spread of early agriculture.

**Figure 2.**
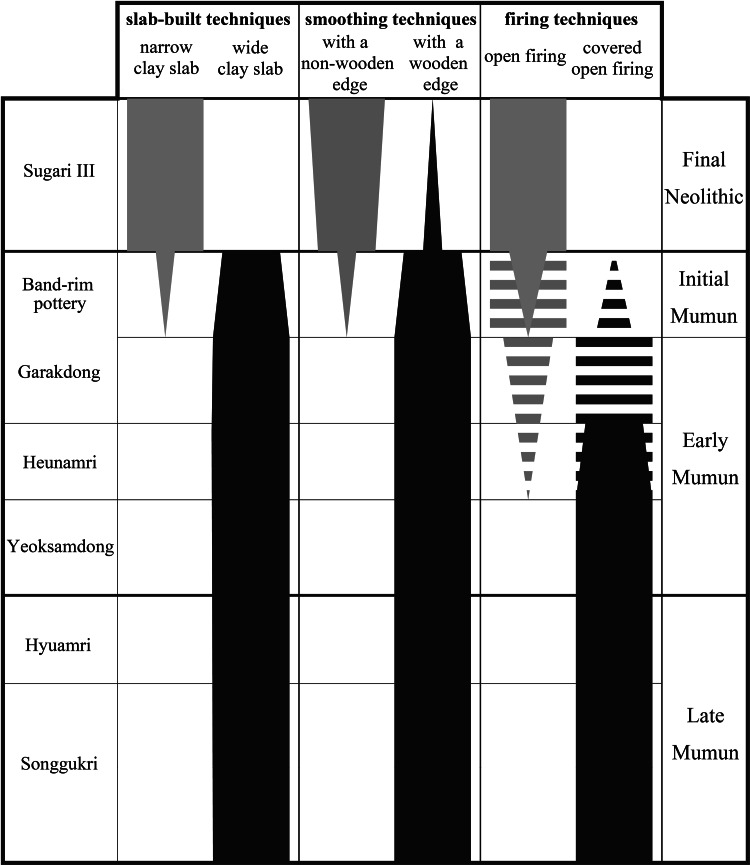
Changes in prehistoric pottery production techniques in the southern Korean Peninsula

The same pottery production techniques existed between Yayoi pottery and the Mumun pottery of southern Korea, primarily in terms of the following four attributes: (a) wide clay slabs; (b) slabs attached on the outer surface of the previous slab; (c) smoothing on the pottery surface with the edge of a piece of wood; and (d) firing in a less elaborate clay kiln on the ground. Mumun pottery culture in the southern Korean Peninsula is divided into three phases: initial, early and late period (Table 2). The initial period is characterised by band-rim pottery, the early period is marked by Garakdon type and Konyol style pottery (Heunamri and Yeoksamdong types) and the late period consists of Hyuamri type and Songgunni type. Kazunori Misaka analysed pottery production techniques between Neolithic pottery and Mumun pottery on the Korean Peninsula (Misaka, 2012). According to his analysis, four attributes in pottery production techniques were seen throughout the Mumun pottery culture, from the initial to the late period (Figure 2). On the contrary, there are no such four attributes in production techniques in Neolithic pottery. Such differences between Neolithic and Mumun pottery are the same as those that existed between Jomon and Yayoi. In this case, the initial period pottery, band-rim pottery, must have spread genealogically from the pottery of other areas. SEM silicon analysis on pottery indicates that rice cultivation in the Korean Peninsula started in the band-rim pottery period of the initial Mumun period (Son et al., 2010). Kim and Park suggested that the migration of rice farmers resulted in language dispersal from northeast China to Korea (Kim & Park, 2020). Therefore, we need to pay attention to how Mumun pottery production styles first started.

**Table 2.** Prehistoric chronology of Northeast Asia

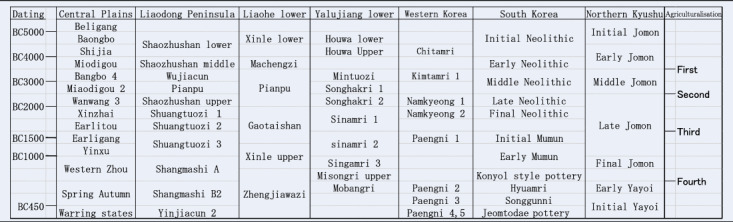

The earliest Mumun pottery in southern Korea is band-rim pottery in the incipient Mumun culture period, from around 1500 BC. Band-rim pottery is thought to be related to the Gonggwiri pottery of the north-central Korean Peninsula (Ahn J., 2010; Bae, 2010), because of similarities in pottery designs and house plans among other aspects. Therefore, Gonggwiri type pottery is believed to be related to band-rim pottery in the incipient.

Mumun culture originated in southern Korea (Miyamoto, 2016, 2017), but the origin of the pottery production techniques of Mumun pottery, such as relatively wide clay slabs, laid with clay slabs attached from the outside and being smoothed with a wooden edge and fired in a primitive clay kiln, was not identified in the Neolithic pottery of Chulmun.

**Figure 3.**
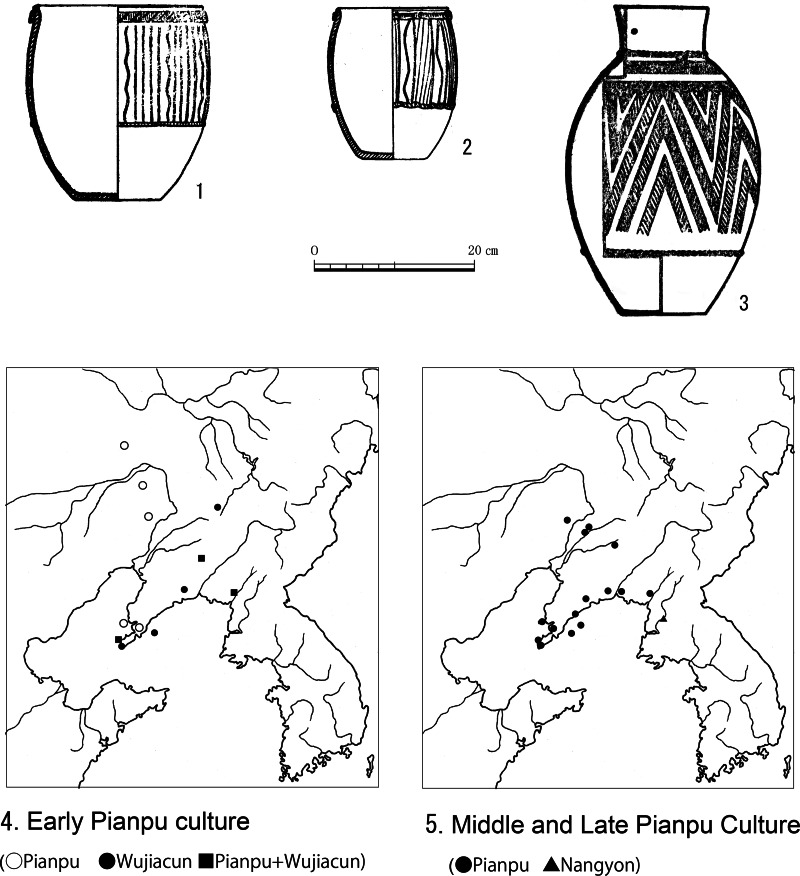
Changes in site distributions in the Pianpu culture

**Figure 4.**
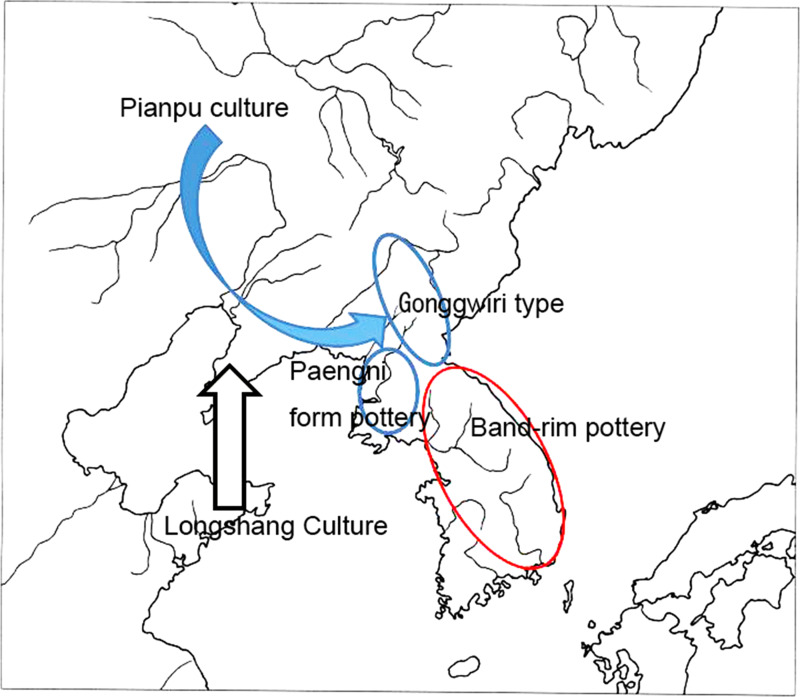
The Pianpu culture and its influence on adjacent areas

**Figure 5.**
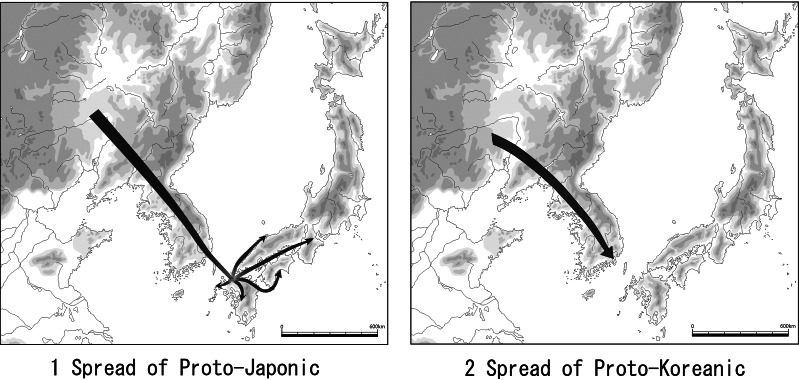
Spread of Proto-Japonic and Proto-Koreanic

On the northwestern Korean Peninsula, the influence of the Pianpu culture (Figure 3-3) in the Liaodong district of Manchuria combined with Namkyeong Chulmun pottery to form the Paengni type of pottery in the Mumun culture (Miyamoto, 2016, 2017). The Pianpu culture is divided into three phases: early, middle and late (Chen & Chen, 1992). The early phase of the Pianpu culture (Figures 3-1 to 3-3) is distributed in the east of Liaoxi district according to the pottery chronology. However, the Wujiacun period of the middle layer of the Shaozhushan culture is distributed in the Liaodong district, peripheral to the distribution of the Pianpu culture (Figure 3-4). The middle and late Pianpu culture replaced the Wujiacun and spread to the Liaodong district and the northwestern Korean Peninsula (Figure 3-5). The Pianpu culture influenced the Chulmun pottery of the northwestern Korean Peninsula, adding a new type of pottery (necked jars) in the Chulmun pottery sequence. The necked jar (Figure 3-3) influenced by the Pianpu culture consisted of Chulmun pottery Namkyeong 1 and Namkyeong 2 of the final Neolithic period on the western Korean Peninsula. This event also accorded with the second stage of agriculturalisation in Northeast Asia in c. 2400 BC (Figure 4). In contrast, in the north-central Korean Peninsula, the influence of the Pianpu culture (Figures 3-1 and 3-2) produced the Gonggwiri pottery type (Dwelling No. 1 at Simgwiri Site) in the middle and upper valley of the Yalu River (Figure 4). This Gonggwiri type directly evolved into the band-rim pottery of the initial Mumun pottery period on the southern Korean Peninsula (Miyamoto, 2016, 2017).

We focused on the production techniques of pottery found at Shangmashi Shell Midden Site on the Liaodong Peninsula, which was excavated by Japanese scholars in 1941 (Miyamoto, 2015b). The analytical results show the existence of four attributes of pottery production skills, such as: (a) slab-built using relatively wide clay slabs; (b) attaching the clay slabs onto the exterior rather than interior surface; (c) smoothing with a wooden edge; and (d) firing the pottery in a less elaborate clay kiln on the ground. It is quite interesting that these four particular attributes of pottery production techniques are limited only to the Pianpu culture period in the chronological scheme from the Neolithic to the early Iron Age (Table 3). Smoothing of the clay surface with a wooden edge appears quite often in Pianpu culture pottery. Smoothing with a wooden edge and the firing technique using a less elaborate clay kiln in the Pianpu culture are also recognised at other sites besides Shangmashi Site. These results indicate that the Pianpu culture is connected with the emergence of new pottery production techniques in the Mumun pottery culture on the southern Korean Peninsula through Gonggwiri-type pottery (Figure 4).

**Table 3.** Changes in pottery production techniques in Shangmashi site, Liaodong Peninsula

Phase/culture	Slab-built techniques						Smoothing techniques				Firing techniques		Blacked surface	Red burnished
	Clayslab			Attaching the clayslab			With a non-wooden edge	With a wooden edge	Rolled stroking	Paddling with parallel lines	Open firing	Covered open firing		
	Narrow	Middle	Wide	Horizontally	From inside	From outside								
Lower Xiaozhushan	●	×	×	●	●	●	●	×	×	×	?	?	×	×
Middle Xiaozhushan	?	?	?	?	?	?	?	?	?	?	?	?	?	?
Wujiacun	–	–	–	–	–	–	–	–	–	–	–	–	–	–
Pianpu	?	?	△	?	?	○	×	●	?	×	?	△	?	●
Upper Xiaozhushan, Shuangtuozi (1)	?	△	△	?	?	○	○	○	●	●	?	?	●	×
Shuangtuozi (2, 3), Upper Shanmashi (Area C)	?	△	△	○	?	●	○	○	●	×	?	?	●	×
Shuangtuozi (2, 3), Upper Shanmashi (Area A Lower)	×	●	×	?	?	●	?	○	●	×	?	?	●	×
Upper Shanmashi (Area A Upper)	×	●	×	?	○	●	?	○	●	×	?	?	●	×
Upper Shanmashi (Area BII)	×	●	×	○	?	●	?	○	●	×	?	?	●	△
Upper Shanmashi (Western Hill)	?	?	?	?	?	?	?	○	●	?	?	?	●	×

●: to exist (accounting for a large percentage), ○: to exist, △: possiblilty to exist, ×: possibility of not existing, ?: unknown, -: not processed to analysis.

Mumun pottery on the Korean Peninsula was established when the pottery styles of the adjacent areas changed to those of the Pianpu culture through the process of diffusion and the influence on these areas by the Pianpu culture. Therefore, new pottery production techniques which were used in Pianpu culture pottery were introduced to Mumun pottery by way of the Pianpu cultural influence on Gonggwiri-type pottery (Figure 4). As such, a pottery style with the same four attributes in terms of production techniques spread from the Pianpu culture in Liaodong to the Mumun culture in southern Korean Peninsula via Gonggwiri type pottery in the northern Korean Peninsula. From there, this pottery style with the same four attributes as the Mumun culture in the southern Korean Peninsula spread to the Yayoi culture in western Japan. The spread of the same genealogical pottery style with these four attributes indicates the spread of the same information via the mediation of language, i.e. Proto-Japonic (Figure 5-1).

However, the transformation of these pottery styles between the Pianpu culture and the band-rim pottery took a significant amount of time – around 1000 years – and the beginning of the Mumun pottery culture started around c. 1500 BC on the southern Korean Peninsula. This is the point at which irrigated agriculture such as rice cultivation along with polished stone tools spread from the Shandong Peninsula to the southern Korean Peninsula by way of the Liaodong Peninsula. Therefore, the establishment of the Mumun pottery culture in southern Korea consisted of a dual situation in that it formed from the foundations of such things as the Pianpu culture of the second stage of agriculturalisation in Northeast Asia, but was established by changes to subsistence activities in the third stage of Northeast agriculturalisation.

Where did Japonic languages originate from? Japonic languages were not coloured by the influence of rice agriculture. Japonic is believed not to have included a vocabulary dedicated to rice (Whitman, 2011). The reason why there was no vocabulary dedicated to rice in Japonic is that Japonic probably originated in eastern Liaoxi. The early Pianpu culture with millet agriculture is mainly distributed in the eastern Liaoxi district (Figure 3-4). Because analysis of pottery production techniques suggests that Mumun pottery originated from the Pianpu culture (Miyamoto, 2016), Japonic is also supposed to have originated from the Pianpu culture (Figure 5-1).

The Mumun culture was subsequently influenced around c. 1500 BC by rice agricultural societies from the Shandong Peninsula through the Liaodong Peninsula, but it is thought that the Mumun continued to speak Japonic. This is during the third stage of agriculturalisation in Northeast Asia. If Japonic languages spread to northern Kyushu via Mumun at the same time as the spread of rice agriculture during the fourth stage of agriculturalisation in Northeast Asia, one can suppose that the transition between Jomon and Yayoi is the same as the transition in language between the traditional Jomon languages and Japonic (Table 1). In this way, when a small number of Mumun culture people immigrated to northern Kyushu and mixed with the Jomon people, Japonic is also believed to have spread to northern Kyushu. Furthermore, the transition between the original Jomon languages and Japonic in northern Kyushu is conjectured to have taken the same amount of time as the transition in style from Yusu 1 to Itazuke 1 pottery. It is also thought that the Jomon languages were replaced by Japonic by way of the same process, as people learned the new pottery techniques discussed above in Japonic, which was spoken by the Mumun people.

## 5. Archaeological explanation for the diffusion theory of the Proto-Koreanic language

Analysis of Shangmashi Shell Midden Site (Miyamoto, 2015b) also indicates that the pottery types changed in the mid-first millennium BC. Long-necked pots and clay-rimmed jars from the Yinjiacun Bronze Age, the sixth and fifth centuries BC, were distributed from Liaoxi to the Liaodong area. This new pottery came from the Liaodong Peninsula into the southern Korean Peninsula and developed into the Jeomtodae pottery (rolled-rim vessels) of the early Iron Age, signifying that people from the Liaodong Peninsula migrated southward into the Korean Peninsula (Miyamoto, 2016, 2017).

The reason why the migrants moved from Liaodong to the Korean Peninsula to establish the Jeomtodae pottery (rolled rim vessel) culture was not the cooler climatic conditions. Rather, the preferred theory is that it was social and political factors in society at the time, as the Yan state, located in the Beijing area, invaded eastward beyond the Yanshan Mountains (Miyamoto, 2017, 2020). The Yan made political contact with chieftains in the eastern Liaoxi district, imposing the hegemony of the Yan state in the sixth and fifth centuries BC. The eastward movement of people from eastern Liaoxi to Liaodong in the Yinjiacun second stage triggered another migration from Liaodong to the Korean Peninsula. In this process, the Yinjiacun second-stage culture spread to the Korean Peninsula and led to the establishment of the Jeomtodae pottery (rolled rim vessel) culture, which included the slender bronze dagger culture at around the fifth century BC. At this time, rice farming settlements disappeared from the archeological record (Ahn S., 2010). This Jeomtodae pottery culture originating from eastern Liaoxi is clearly not associated with wet rice agriculture but with millet and wheat agriculture.

On the other hand, Hudson and Robbeets insisted that Proto-Macro-Koreanic spread before Proto-Japonic from the Liaodong Peninsula to the Korean Peninsula with millet agriculture around 3500 BC (Hudson & Robbeets, 2020). However, millet agriculture with Chulmun pottery and agricultural stone tools spread from the northwestern and middle western Korean Peninsula to the southern Korean Peninsula in the place of Yunggimun pottery culture around 3500 BC (Miyamoto, 2014, 2017). Millet agriculture with agricultural stone tools spread from Liaoxi to the Liaodong Peninsula and the western Korean Peninsula in the fifth millennium BC (Li et al., 2020). In addition, pottery styles clearly differ between the Liaodong Peninsula with Xiaozhushan lower layer pottery and the Korean Peninsula with Chulmun pottery (Kim & Park, 2020). Therefore, it is difficult to conceive that the theory of a dispersal of language from the Liaodong Peninsula to the Korean Peninsula is archaeologically connected with the spread of Chulmun pottery with agricultural stone tools to southern Korea in the fourth millennium BC.

The Koreanic language is believed to have spread with this migration to the Korean Peninsula to form the slender dagger (Korean type dagger) culture (Whitman, 2011). This cultural influence from eastern Liaoxi district to the Korean Peninsula indicates the spread of Proto-Koreanic, with people fleeing owing to the continuing territorial threat posed by the Yan state during the Warring States period of China (Miyamoto, 2017, 2020). This cultural spread originated from the Liangquan Culture or the Yinjiacun second stage in the Liaodong district in Manchuria. Because the early Iron Age is associated with the spread of Koreanic, it is assumed that the birthplace of the Koreanic language was also in eastern Liaoxi district (Figure 5-2). The Jeomtodae pottery culture continued to develop into the forms familiar during the Proto-Three Kingdoms culture, directly changing to the Three Kingdoms. The language in Silla of the Three Kingdoms was surely Korean (Robbeets, 2020). Therefore, Proto-Koreanic was spoken in the Jeomtodae pottery culture period. Jeomtodae pottery culture was not genealogically connected with Mumun culture.

As such, it is assumed that Proto-Japonic was replaced by Proto-Koreanic in the early Jeomtodae pottery culture period. Proto-Japonic was spoken in the Korean Peninsula and spread to the Japanese archipelago at the beginning of the Yayoi culture. This replacement of one language with another accords with Unger's idea that Proto-Korean spread from northeast China to the Korean Peninsula in the Early Iron Age of Korea (Unger, 2014). On the other hand, Kim and Park cast doubt on Whitman's suggestion (2011) that the Proto-Koreanic language entered along with the slender daggers (Kim & Park, 2020). The Jeomtodae pottery culture in Liaoxi and Liaodong of northeast China with Liaoning-type daggers spread to the Korean Peninsula, where slender daggers developed independently (Miyamoto, 2020). Then Jeomtodae pottery culture produced iron tools influenced by the Chinese Yan states and the Lelang administration office of the Han period in the Proto-Three Kingdom period. Therefore, it is reasonable to assume that the people of the Jeomtodae pottery culture, the direct ancestors of Three kingdom states, spoke Proto-Koreanic.

## 6. Conclusion

Linguistic research suggests that Proto-Japonic and Proto-Koreanic split off from the Transeurasian languages in southern Manchuria (Unger, 2014; Robbeets et al., 2020). Proto-Japonic first spread to the Korean Peninsula from southern Manchuria and then to northern Kyushu at the beginning of the Yayoi culture. Proto-Koreanic spread at a later date to the Korean Peninsula from southern Manchuria, gradually driving out Proto-Japonic.

The potteries of the Pianpu, Mumun and Yayoi cultures is supposed to be genealogically linked to each other across different ages, especially with the same pottery production techniques exhibiting the following four attributes: wide clay slabs, slabs attached on the outer surface of the previous slab, smoothing on the pottery surface with the edge of a piece of wood and firing in a less elaborate clay kiln on the ground. These same pottery production techniques are thought to have been handed on through language. This is the reason why the genealogical sequence of the Pianpu, Mumun and Yayoi cultures indicates the spread of Proto-Japonic. Therefore, the Proto-Japonic of the Pianpu culture originated from the eastern Liaoxi district or Liaohe basin in southern Manchuria at around 2700 BC, and spread to the Mumun culture in the southern Korean Peninsula via the Gonggwiri type pottery in the northern Korean Peninsula at around 1500 BC (Figure 5-1).

Proto-Japonic reached northern Kyushu in the ninth century BC with the spread of irrigated rice agriculture, and completely replaced the Jomon languages during the Itazuke pottery period in northern Kyushu in the sixth to fifth centuries BC. As a process, this replacement of languages took place over a period of around 300 years. In this case, the people did not necessarily change in terms of their genetic makeup. The Japonic language-speaking people, who accompanied the Itazuke type pottery, came from the Fukuoka Plains and migrated into western Japan, where Japonic gradually replaced the local Jomon languages during the early Yayoi period (Figure 5-1). After the Middle Yayoi period, which started at the beginning of the third century BC, Yayoi culture became radically different from the Jomon pottery tradition that still existed in eastern Japan. Despite this, Proto-Japonic languages are thought to have gradually replaced the local Jomon languages in these areas from the Late Yayoi to the beginning of the Kofun period, second to third centuries AD.

In the sixth and fifth centuries BC, the Yan states of the Eastern Zhou period in China extended their territory across the Yanshan Mountains and politically influenced chieftains in Liaoxi district. From this time, migrants of the Yinjiacun second stage made their way from eastern Liaoxi to Liaodong and then on to the Korean Peninsula at around the fifth century BC, where they established the Jeomtodae (rolled rim) pottery. The Jeomtodae pottery itself started at the second and third phases of Shuangtuozi period, the latter half of the second millennium BC in Liaodong Peninsula. The spread of rolled rim vessels from eastern Liaoxi to the southern Korean Peninsula indicates the route of the Proto-Koreanic (Figure 5-2).

The homeland of both languages is the same based on archeological evidence, and they are kindred language families. However, the time difference in dispersal between the two languages in Northeast Asia is about 1000 years and the spread of both languages was not related to the demic diffusion of agriculture, except for the fourth spread of agriculture in Northeast Asia at the beginning of Yayoi culture in northern Kyushu. This dispersal hypothesis for Proto-Japonic and Proto-Koreanic based on archeological explanations does not necessarily disturb the linguistic hypothesis that Proto-Japonic and Proto-Koreanic split off from the Transeurasian languages in southern Manchuria (Unger, 2014; Robbeets et al., 2020). Rather the dispersal hypothesis supports the linguistic hypothesis.

## Data Availability

All data used for this article can be found in the cited literature.
